# Supplementary material for: Concordance among patients and physicians about their ideal of autonomy impacts the patient-doctor relationship: A cross-sectional study of Mexican patients with rheumatic diseases
Source: PLoS One. 2020 Oct 29;15(10):e0240897. doi: 10.1371/journal.pone.0240897 (PMC7595407; doi:10.1371/journal.pone.0240897)
Supplement: S1 Appendix — Consecutive steps/criteria for item´s reduction N° (%) of patients with at least one visit to the outpatient clinic with the 10 most frequent diagnosis specified. (PDF) [file pone.0240897.s001.pdf]

Supplementary table 1. N° (%) of patients with at least one visit to the outpatient clinic with the 10 most frequent diagnosis specified (January 2018 to December 2018, N=5000 patients).

**Specific diagnosis[1]**	**Nº (%) of patients**
SLE	1652 (33.0)
RA	1578 (31.6)
Sclerodermia	239 (4.8)
Systemic Vasculitis (SV)	220 (4.4)
Primary Sjögren Syndrome (PSS)	190 (3.8)
Spondyloarthropaties (SA)	174 (3.5)
Inflammatory Miopathies (IM)	150 (3.0)
Primary Anti-Phospholipid Syndrome (PAPS)	150 (3.0)
Mixed Connective Tissue Disease (MCTD)	94 (1.9)
Adult Still disease	29 (0.58)
Other diagnosis	524 (10.5)

[1]Diagnosed based on the attending rheumatologist criteria. SLE=Systemic Lupus Erythematosus. RA=Rheumatoid Arthritis.
