# Supplementary material for: Concordance among patients and physicians about their ideal of autonomy impacts the patient-doctor relationship: A cross-sectional study of Mexican patients with rheumatic diseases
Source: PLoS One. 2020 Oct 29;15(10):e0240897. doi: 10.1371/journal.pone.0240897 (PMC7595407; doi:10.1371/journal.pone.0240897)
Supplement: S2 Appendix — Summary of the cultural adaptation and translation process. (PDF) [file pone.0240897.s002.pdf]

Supplementary table 2. Summary of the cultural adaptation and translation process.

STEPS	MODIFICATIONS AND INCORPORATIONS
Preparation	Two local collaborators were recruited; one of them was bilingual (Spanish, Dutch). In addition, the IPAs (Dutch version) developer (ACM) was invited and agree to participate.
Forward translation	Two independent forward translations were received (Dutch-Spanish).<br>Translations were provided by certified translators.
Reconciliation of the forward translation into a single forward translation	Thirteen bioethicists were invited to participate; eleven agreed to evaluate items and instructions from both translations.<br>A first version (v1) was integrated according to their evaluation and approved by the local bilingual collaborator.<br>The v1 was adapted to rheumatic diseases by a rheumatologist and bioethicist, in charge of the project (VPR) and v2 was obtained.
Back translation	One backward translation into the source language (Dutch) was performed by a certified translator.
Back translation review	The local collaborator and the IPAS developer approved the back translation to ensure the conceptual equivalence of the translation.
Harmonization	The project responsible (VPR) did not identify potential problematic items.
Congnitive debriefing	The v2 was evaluated by 13 outpatients from the outpatient clinic of the Department of Immunology and Rheumatology (4 patients had SLE, 3 had RA, 2 each had MCTD and Sclerodemia and 1 patient each had SV and Adult Still Disease).<br>Two patients recommended minor changes in the instructions, 4 patients changes in item coded as 11, 2 patients changes in item coded as 19 and 1 patient each, suggested changes in items coded as 14 and 18, respectively.
Review of cognitive debriefing results and finalization	The project responsible incorporated all the suggestions but 1, which was considered conceptually inadequate. This version (v3) was revised and approved by the local collaborator.
Proofreading	The v3 was proofread by the project responsible, the local collaborator and an independent reviewer from the Department of Immunology and Rheumatology.
Final report	Elaborated by the responsible of the project.

SLE=Systemic Lupus Erythematosus; RA=Rheumatoid Arthritis; MCTD=Mixed Connective Tissue Disease; SV=Systemic Vasculitis.
