# Supplementary material for: Concordance among patients and physicians about their ideal of autonomy impacts the patient-doctor relationship: A cross-sectional study of Mexican patients with rheumatic diseases
Source: PLoS One. 2020 Oct 29;15(10):e0240897. doi: 10.1371/journal.pone.0240897 (PMC7595407; doi:10.1371/journal.pone.0240897)
Supplement: S3 Appendix — Simple linear regression analysis, to ascertain the magnitude of the relationship between PDRQ-9 score and patient-doctor encounters with concordance in the ideal of autonomy, in 4 rheumatic conditions. (PDF) [file pone.0240897.s003.pdf]

Supplementary table 3. Simple linear regression analysis, to ascertain the magnitude of the relationship between PDRQ-9 score and patient-doctor encounters with concordance in the ideal of autonomy, in 4 rheumatic conditions.

	RA and other synovial disorders, N=165	Connective Tissue Disorders, N=277	Vasculitides, N=33	Spondyloarthritis, N=22
N° (%) of patient-doctor encounters with concordance	211 (76.2)	116 (70.3)	22 (66.7)	14 (63.6)
β coefficient	0.363	0.388	0.384	0.073
95%CI	0.111 to 0.615	0.087 to 0.689	-0.305 to 1.072	-0.74 to 0.886
p value	0.005	0.012	0.264	0.853
R^2	0.028	0.038	0.040	0.002
