# Supplementary material for: Concordance among patients and physicians about their ideal of autonomy impacts the patient-doctor relationship: A cross-sectional study of Mexican patients with rheumatic diseases
Source: PLoS One. 2020 Oct 29;15(10):e0240897. doi: 10.1371/journal.pone.0240897 (PMC7595407; doi:10.1371/journal.pone.0240897)
Supplement: Attachment — Submitted filename: Response to reviewers.docx [file pone.0240897.s004.docx]

**RESPONSE TO REVIEWERS**

**JOURNAL REQUIREMENTS**

**1. Please ensure that your manuscript meets PLOS ONE's style requirements, including those for file naming. The PLOS ONE style templates can be found at**

[**https://journals.plos.org/plosone/s/file?id=wjVg/PLOSOne_formatting_sample_main_body.pdf**](https://journals.plos.org/plosone/s/file?id=wjVg/PLOSOne_formatting_sample_main_body.pdf) **and**

[**https://journals.plos.org/plosone/s/file?id=ba62/PLOSOne_formatting_sample_title_authors_affiliations.pdf**](https://journals.plos.org/plosone/s/file?id=ba62/PLOSOne_formatting_sample_title_authors_affiliations.pdf)

*Response: We have revised PLOS ONE´s manuscript style requirements.*

**2. Please provide additional details regarding participant consent. In the ethics statement in the Methods and online submission information, please ensure that you have specified how verbal consent was documented and witnessed.**

*Response: We have updated the ethics statement in Methods section and in the online submission.*

**3. Please amend your authorship list in your manuscript file to include author** [**Gregorio Tomás Obrador**](mailto:gobrador@up.edu.mx)**.**

*Response: We have amended the authorship list.*

**COMMENTS TO THE AUTHOR**

**Reviewer #1**: In this interesting article Pascual Ramos and colleagues investigated the concordance among patients and physicians about their ideal of autonomy impacts the patient-doctor relationship. The article is well written and provides interesting insights in the topic.

*Response*: We appreciate the reviewer´s comments.

My major concerns relate to:

**-Gender bias: Almost all patients included in all phases of the study are females and although this reflects the prevalence of the included diseases (e.g. SLE, RA), this necessarily hampers the generalizability of the results. This in fact prevents the assessment of whether gender is a factor that should be considered in regression models.**

*Response: We agree with the reviewer and have added as a limitation of the study. In addition, we have performed two different analysis, and updated the corresponding paragraph; in the first one, only variables with statistical significance in the univariate analysis were included into the models; in the second analysis, female sex and age were additionally forced into the model, as both had been associated to share-decision models in some populations (an additional reference is provided, number 31); importantly, with both analysis same results were obtained.*

**-Imbalance of disease types: SLE and RA account for more that half of the sample so again the question remains on how these results can be generalised to all other diseases that are poorly represented in the sample. Perhaps it would make sense to pool patients by disease groups (e.g. inflammatory arthritis vs connective tissue diseases, ideally by including additional patients to balance the numbers). This would also allow to unmask disease-related differences.**

*Response: We agree with the reviewer; our population is highly represented by SLE and RA patients (probably due to local reference bias); we have added in the manuscript table 7, which summarizes a similar distribution of preferred autonomy ideal among the different rheumatic diseases. In addition, we have addressed in the limitation section, that the imbalance observed, prevents results generalization; we also propose a patient´s redistribution into 4 categories (a reference is provided) and show the results in supplementary table 3.*

**Reviewer #2**: The authors found that concordance of autonomy ideal among patients and their rheumatologists positively impacts on the patient-doctor relationship.

The study also revealed that Mexican patients and rheumatologists ideal of autonomy was primarily physician-centered or paternalistic.

This is an interesting study that reveals that the concordance about ideal autonomy improves patient - doctor relationship in Mexican patients with rheumatic diseases.

In literature is reported that patients’ preferences and autonomous decision-making are influenced by demographic variables (with younger ,better educated patients and women being quite consistently found to prefer a more active role in decision making), their experience of illness and medical care, their diagnosis and health status, the type of decision they need to make, the amount of knowledge they have acquired about their condition, their attitude towards involvement, and the interactions and relationships they experience with health professionals. Their preferences are likely to develop over time as they gain experience and may change at different stages of their illness.

I think that this study enriches the knowledgment about clinical decision making in a specific geographic area.

I appreciated methodological steps and interdisciplinary collaboration.

I have not any major or minor remarks

*Response: We highly appreciate the reviewer´s comments.*
